# Automated thermo-mechanical therapy for immediate relief in chronic non-specific lower back pain: a randomized controlled trial

**DOI:** 10.3389/fnrgo.2025.1674928

**Published:** 2026-01-09

**Authors:** Kyle Donnery, Giuseppina Pilloni, Mohamad FallahRad, Kiwon Lee, Byungyun Han, Soonhi Park, Jihye Kim, Leigh Charvet, Marom Bikson

**Affiliations:** 1 Department of Biomedical Engineering, The City College of New York, New York, NY, United States; 2 Department of Neurology, NYU Grossman School of Medicine, New York, NY, United States; 3 Clinical Research Institute, Ceragem Clinical Inc, Gyeonggi-do, Seoul, Republic of Korea

**Keywords:** ATT, cNSLBP, pain, randomized controlled trial (RCT), thermo-mechanical therapy

## Abstract

**Objective:**

Chronic non-specific lower back pain (cNSLBP) is a prevalent and disabling condition, imposing a substantial socioeconomic burden due to high healthcare costs and productivity losses, with limited accessible and effective long-term treatment options. Automated Thermo-mechanical Therapy (ATT) is a promising, non-drug intervention that leverages innovative technical advances to provide multimodal pain relief, offering accessibility and low-cost delivery. This study tested ATT for immediate pain relief in individuals with cNSLBP in a single-session, double-blind, randomized controlled trial.

**Methods:**

Forty participants with cNSLBP were assigned to receive either active ATT (*n* = 20) or control ATT (*n* = 20) in a 40-min session with urn randomization. The active device applied heated cylindrical rollers along the spine, using far-infrared heat and mechanical tissue stimulation tailored to spinal alignment. In the control condition, the device used minimal mechanical therapy intensity without heat, targeting only the cervical area to avoid lower back therapeutic effects. Pre- and post-intervention assessments measured changes in pain intensity (primary outcome) via a 100-mm Visual Analog Scale for Pain (VAS-P100), alongside secondary outcomes assessing pain characteristics, anxiety, and functional mobility.

**Results:**

The active ATT group showed a significant reduction in pain on the VAS-P100, with an average decrease of 46.8%, compared to 17.0% in the control group. Participants in the active group also reported significantly greater subjective pain relief (*p* = 7.88e−05). Secondary outcomes demonstrated significant improvements in lumbar flexibility (Modified-Modified Schober Test, MMST) for the active ATT group compared to the control group (*p* = 0.0031). No adverse events were reported, and all participants tolerated the intervention well.

**Conclusions:**

A single session of ATT provides immediate, significant pain relief in individuals with cNSLBP, supporting its potential as a safe, non-invasive option for managing chronic back pain. Future studies should examine the long-term benefits of repeated ATT sessions and explore mechanistic insights into thermo-mechanical stimulation's effects on pain and function.

**Clinical Trial Registration:**

ClinicalTrials.gov, identifier: NCT06769321.

## 1 Introduction

Chronic non-specific lower back pain (cNSLBP) is persistent pain localized from the costal margin to the inferior gluteal fold that lasts for 12 weeks or longer and lacks a specific identifiable pathology (Maher et al., 2017). CNSLBP affects approximately 4–20% of the global population and is especially prevalent among adults aged 30–60 (Maher et al., 2017; Buchbinder et al., 2018) years. This often debilitating condition imposes a significant socioeconomic burden, impacting individuals during their peak working years. As a leading cause of disability worldwide, cNSLBP affects millions and contributes to substantial healthcare costs and productivity losses. cNSLBP has been challenging to treat with medical interventions alone because of the complex interaction of biological, psychological, and social factors that influence its onset and persistence.

Pharmacological treatments for cNSLBP, including NSAIDs, opioids, and antidepressants, demonstrate mixed efficacy in short-term pain reduction. However, their long-term effectiveness is limited or complicated by potential risks such as dependency and adverse side effects, underscoring the need for a multimodal approach that incorporates non-pharmacological therapies (Thompson et al., 2020).

Non-pharmacological interventions, such as exercise, psychological therapies, acupuncture, and manual therapies, show consistent benefits in improving pain and function, particularly over the long term. These approaches are generally safer and may offer more sustainable outcomes compared to pharmacological treatments, including for cNSLBP management (Jiang et al., 2022; Deyo et al., 2015; Tetsunaga et al., 2018). However, the effectiveness of these approaches depends on accessibility (particularly in underserved areas), costs (insurance coverage), patient adherence, and quality and consistency of manual intervention delivery. There is a need for a convenient and accessible treatment alternative that requires minimal active effort from participants and produces reliable acute relief for patients.

Automated Thermo-mechanical Therapy (ATT) fills this need as a neuroergonomic intervention that uses an automated device combines mechanical pressure and heat to target musculoskeletal pain and stiffness. This approach can improve the range of motion and functional mobility, making it particularly useful for cNSLBP and related conditions. ATT is delivered through freestanding devices that allow for tailored, consistent treatment sessions with minimal therapist involvement, it achieves this through scans powered by adaptive sensors (Buselli et al., 2011). The automation in these human-centric devices ensure uniform application of heat and mechanical pressure, optimizing therapeutic outcomes by maintaining consistent quality across sessions while reducing the need for continuous manual adjustments by a therapist. Advanced ATT is delivered by a device that includes multi-axis traction and far-infrared thermal projectors (Kim et al., 2022). In this manner, the delivery of the therapy can be both individualized to a patient's unique anatomy and delivered in consistent automated doses.

The mechanism of ATT's therapeutic benefits include enhanced blood circulation, muscle relaxation, and reduced pain through the combined effects of heat and mechanical stimulation. Heat therapy has long been recognized for its analgesic effects, particularly in relation to musculoskeletal pain (Chen et al., 2015; Nadler, 2015). The application of heat to the skin has been shown to promote vasodilation and increase blood flow. This provides increased oxygen and nutrients to the affected tissues (Rabkin, 1987; Hardy and Woodall, 1998). It has also been shown to increase local rates of metabolism and removal of metabolic waste products (Nadler, 2015), as well as increase the elasticity of muscle tissue potentially increasing muscle flexibility and relaxation, reducing tension and stiffness, contributing to pain relief, and improving mobility (Hardy and Woodall, 1998). Heat therapy also activates thermoreceptors, in particular, transient receptor potential vanilloid 1 (TRPV1) receptor, which, in the context of lower back pain and gate control theory, activates signals that block the processing of pain signals in the lumbar dorsal fascia and spinal cord (Freiwald et al., 2021; Green, 2004).

Chronic non-specific lower back pain (cNSLBP) encompasses a wide range of etiologies, with mechanical factors including muscle strain, nerve compression, intervertebral disc herniation, and degenerative changes (Emorinken et al., 2023). Individuals with musculoskeletal disorders often exhibit increased tension in the musculature surrounding the affected area, and such muscle spasms may contribute to secondary lumbar pain (Hirayama et al., 2006). To address these symptoms, various physical therapeutic modalities—such as thermotherapy, massage, manipulation, pharmacologic interventions, injections, and biofeedback—have been employed (Roland, 1986). Among these, physical therapy and rehabilitative approaches are recognized as effective non-pharmacological strategies for reducing pain and muscle tension while promoting spinal functional recovery. These modalities are particularly beneficial for elderly patients and individuals with chronic conditions for whom pharmacologic treatment options may be limited (Karateev et al., 2022). The Automatic Thermal Massager (ATT) developed by Ceragem is designed based on these principles of physical therapy and is anticipated to facilitate the relaxation of paraspinal muscle stiffness and tension, thereby contributing to the alleviation of muscle-related pain associated with LBP and supporting spinal functional restoration (Emorinken et al., 2023).

Mechanical stimulation involves the application of controlled and rhythmic pressure strokes to the body, mimicking the effects of manual massage therapy. ATT typically uses spinal traction and mechanical rollers to apply this form of therapy. Mechanical stimulation-based therapies are primarily used to manage pain (Kim et al., 2020, 2022; Choi et al., 2020a), promote relaxation/autonomic regulation (Lee et al., 2011), increase blood flow (Lee et al., 2011; Tiidus and Shoemaker, 1995; Sefton et al., 2010), and reduce inflammation (Rodrigues et al., 2020). Spinal traction is used for the treatment of an array of intervertebral disc-related problems, pinched nerves, and lower back pain.

While heat and mechanical stimulation individually show promise in alleviating musculoskeletal pain, their combined application in a single therapeutic session may provide synergistic effects. Synergistic effects are expected, particularly, both interventions have been associated with increased blood flow. Additionally, biomechanically, heated tissue has been shown to have increased malleability, which allows for mechanical stimulation to have greater deflection of the tissue. Furthermore, by using mechanical stimulation to deflect the tissue and decrease the distance between the target tissue and the thermal actuator, the target tissue receives more thermal energy.

This dual-action approach holds the potential to enhance treatment outcomes for patients with cNSLBP by addressing multiple underlying mechanisms of pain and dysfunction. To address the need for a device capable of providing acute pain relief for cNSPLBP, a single session immediate pain relief model was chosen. Here, we test acute pain relief and functional improvements following a single session of ATT in individuals with cNSLBP.

## 2 Methods

### 2.1 Trial design

**Figure 1:**
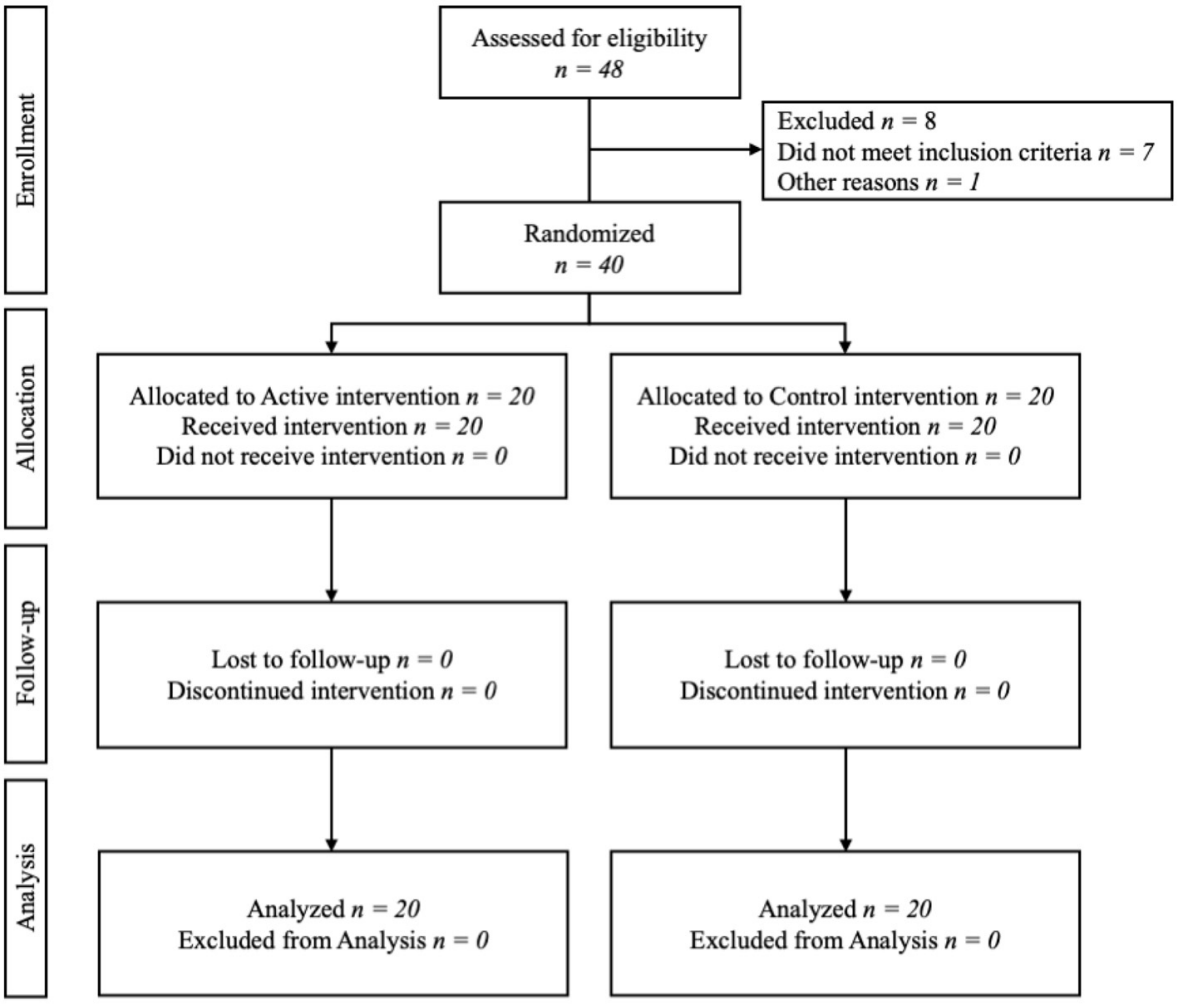
Flow of participants in a study of the effect of ATT on cNSLBP.

This randomized controlled trial (RCT) used a single-session, double-blind, parallel-arm design in accordance with CONSORT guidelines (Figure 1). The administration of the ATT intervention was 40 min, and the research visit lasted approximately 2.5 h in total including with pre- and post-intervention assessments. Participants were randomized to either active or control ATT arm by an independent study team member who was not involved in outcome assessment or intervention delivery using urn randomization. This was done using forty folded paper strips put in a sealed obtuse container. Twenty strips had the serial number of the control device and twenty had the serial number of the active device, one strip was pulled for each participant. Devices used in the study were preprogrammed by the manufacturers to deliver the specified active or control intervention. As this was a pilot study, no formal *a priori* sample size calculation was performed. The sample size of 40 participants was chosen in line with recommendations for pilot studies, which typically include 24–50 participants to estimate feasibility and variability for future trials (Julious, 2005; Sim and Lewis, 2012).

The study received ethical approval from the City College of New York Institutional Review Board (2024-0288). This study was registered at ClinicalTrials.gov (NCT06769321). All eligible participants provided written informed consent before completing any research procedures.

### 2.2 Participants

Individuals self-reporting cNSLBP were recruited through regional advertisements and community networks of the City University of New York City College campus. Interested participants were first screened by phone. Eligibility criteria included ages 18–65 years and cNSLBP persisting for ≥12 weeks. Additionally, participants needed a conversational level of English to engage in study procedures. Potential participants were excluded if they had contraindications to mechanical manipulation of the back; history of back surgery within the past three years; recent back injury; presence of back implants or active implanted devices; low back pain resulting from other specific conditions (e.g., infection, neoplasm); sensitivity to heat; circulatory insufficiency; heart disease; or being pregnant or breastfeeding. Individuals with a history of substance use disorder or those with a body weight over 298 lbs (135 kg) were also excluded

Potential participants who met the initial screening criteria were next scheduled for a study visit. When participants arrived for the session, informed consent was obtained before any study procedures were carried out. They then completed additional eligibility criteria including currently rating their pain rating to be at least 40 mm on a 100-mm Visual Analogue Scale for Pain (VAS-P100).

### 2.3 Equipment

**Figure 2:**
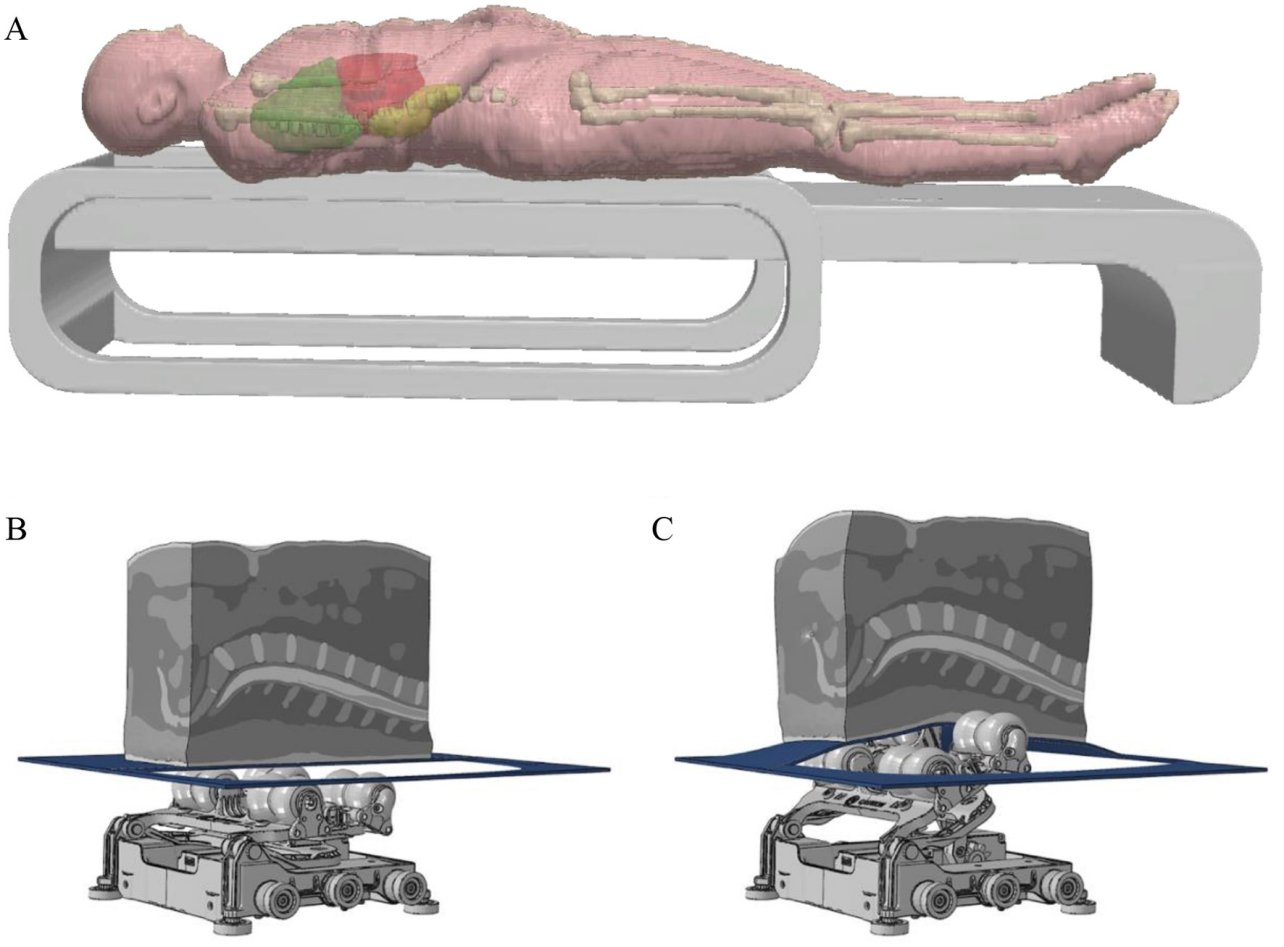
Model of mechanisms for delivery of ATT to the spinal column. **(A)** Model of automated thermo-mechanical therapy device, the thermal projectors and cylindrical rollers are housed in the superior portion of the device, where the vertebral column rests. **(B)** Model of the internal mechanical components of the device resting beneath the spine and spinal tissue when inactive. **(C)** Model of the internal mechanical components of the device when activated, depicting deflection of the spine and spinal tissue.

**Figure 3:**
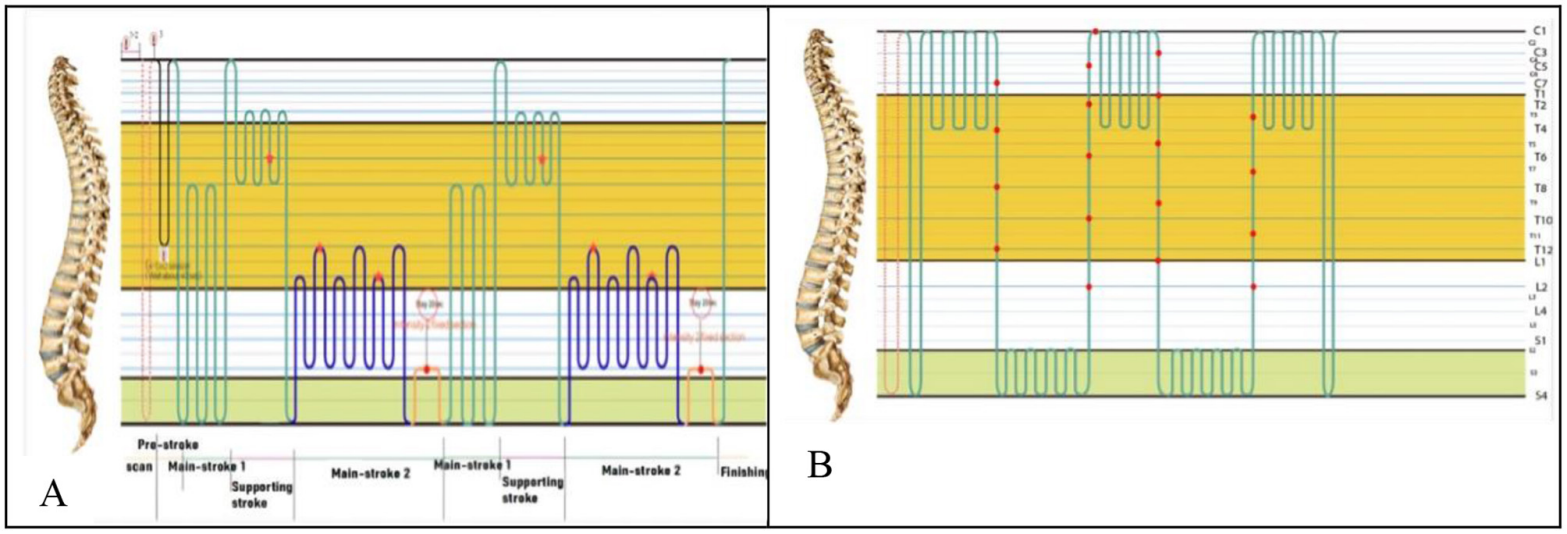
ATT protocols for active and control conditions. **(A)** The program includes scan, pre-stroke, main-stroke1, supporting-stroke, main-stroke2 and finish-stroke cycles. It uses the automatic mode, which measures the length of the spine according to each cycle zone and is set to apply the in-device temperature and level (range: 30–65 °C/Level: 1–9). After the scan, in the pre-stroke phase, breathing through verbal cues is utilized to facilitate relaxation of deep muscles in the lumbar region. During the main-stroke1 and supporting-stroke, gentle massage is applied to the muscles causing lower back pain. In main-stroke2, intensity is increased and speed decreased to concentrate more intensely on the major muscles contributing to lower back pain. The 20-s stay maneuver in main-stroke2 encourages pelvic stabilization for balancing the muscles connected to the pelvis. This thermal massage cycle is repeated twice, totaling 40 min of thermal massage. To enhance the synergistic effect of pain relief during the thermal massage, an external projector is placed on the abdomen at 30–60 °C. **(B)** The comparator device is a sham device that has the same outer appearance as the investigational device. Throughout the entire massage session, the intensity was set to a low level, below level 1, in areas unrelated to where the major muscles causing lower back pain are located, minimizing the actual massage effect. Additionally, the temperature was turned off to exclude the effect of thermal therapy.

The study intervention utilized two MASTER V6 ATT devices (Master series, CGM MB-1701, CERAGEM Co. Ltd., Cheonan, Korea; Figures 2A, B), each programmed for either active or control treatment. The device programmed for active ATT device (Figure 3A) used heated cylindrical rollers that moved along the spine, applying tissue stimulation and emitting far-infrared heat, which penetrated muscle tissue to promote circulation and muscle relaxation. The device's body scanning system mapped each individual's spine using weight and current sensing sensors, adjusting roller positions to align with spinal length and curvature for precision and personalization. The active program includes scan, pre-stroke, main-stroke1, supporting-stroke, main-stroke2 and finish-stroke cycles. The roller temperature was adjustable, with settings ranging from 30 to 65 °C, tailored to each participant's comfort. The mechanical intensity was also adaptable, focusing on the lower back to ensure a targeted therapeutic effect. In contrast, the control device (Figure 3B) used for the control group delivered a minimal mechanical therapy, intensity below level 1, with no heat, and the mechanical therapy was directed primarily to the cervical area, distant from the lower back, to minimize any therapeutic effect (Figure 3B).

### 2.4 Procedures

Once enrolled and consented, participants were randomly assigned to receive either an active or control ATT condition. To ensure double blinding, participants were assigned to a In the active ATT group, participants completed a 40-min session involving various therapy phases, with adjustable intensity and heat applied to the lower back (Figure 2A). The thermal settings ranged from 30 to 65 °C, adjusted based on individual preference, while mechanical intensity was modified to ensure participant comfort. In contrast, participants in the control ATT group received a control session with mechanical intensity set below level 1 and no heat applied (Figure 2B). Additionally, the mechanical stimulation primarily targeted the cervical area, distant from the lower back, to prevent any specific therapeutic effects (Figure 2C).

### 2.5 Automated thermo-mechanical therapy device

The MASTER V6 (Master series, CGM MB-1701, CERAGEM Co. Ltd., Cheonan, Korea) used in this study is a commercially available automated thermo-mechanical therapy device that combines mechanical spinal stimulation with far-infrared (FIR) heat treatment (see Figure 3). It delivers ATT through the Ceracore Engine™, which integrates the proprietary Spine-TECH™ and Thermal-TECH™ technologies. The device delivers the intervention through heated rollers and an automated body scanning system. The cylindrical rollers move along the spine's natural curvature, applying mechanical tissue stimulation to promote circulation and muscle relaxation. The rollers emit far-infrared (FIR) heat, which penetrates muscle tissue to improve local blood flow. The device's body scanning system uses weight and current sensing sensors to map each individual's spine, adjusting the roller positions based on spinal length and curvature for precise, personalized stimulation. The temperature of the rollers can be customized for specific therapeutic needs, ensuring consistent heat delivery.

### 2.6 Outcome measures

Study outcomes were administered before and immediately after the 40-min active or control intervention. The primary outcome was change in pain intensity, measured using the 100-mm VAS-P (VAS-P100), before and after the intervention. The VAS-P100 is a unidimensional measure of pain intensity, used to record patients' pain progression or compare pain severity between patients. The 100-mm VAS-P is a straight horizontal line of fixed length of 100 mm. The ends are defined as the extreme limits of the parameter to be measured (pain) orientated from the left (best) to the right (worst). The participant marks on the line the point that they feel represents their perception of their current state. The score is determined by measuring in millimeters from the left-hand end of the line to the point that the patient marks (Jensen et al., 2003).

Secondary outcomes focused on assessing disability related to back pain, pain characterization, anxiety, pain relief, and functional mobility. These were measured by:

Self-reported Questionnaires:

- Roland-Morris Disability Questionnaire (RMDQ): the Roland-Morris is a 24-item self-report questionnaire about how low-back pain affects functional activities. Total score can range from 0 (no disability) to 24 (severe disability). The RMDQ is scored by adding up the number of items the patient checks. (Administered only at the pre-ATT intervention to characterize how low back pain affects on daily functions; Roland and Morris, 1983).
- McGill Pain Questionnaire (MPQ): this questionnaire is used to evaluate a person experiencing significant pain, to monitor the pain over time and to determine the effectiveness of any intervention. The questionnaire evaluates three different domains: (1) What does the pain look like?; (2) How does your pain change with time?; (3) How strong is your pain? (Melzack, 1975).
- State-Trait Anxiety Inventory (STAI): this questionnaire is widely used to measure the state and trait components of anxiety (Spielberger, 1983).
- 5-point Verbal Rating Scale for Pain Relief (VRS): verbal rating scales about pain relief on a 5-point rating scale: none = 0, slight = 1, moderate = 2, good = 3, complete relief= 4 (Administered only at the post-ATMB intervention; Ferreira-Valente et al., 2011).

Functional Tests:

- Modified-Modified Schöber Test (MMST): this is a physical examination used to measure the ability of a patient to flex the lower back. The patient is standing, examiner marks both posterior superior iliac spine (PSIS) and then draws a horizontal line at the center of both marks. A second line is marked 15 cm above the first line. The patient is then instructed to flex forward without bending the knees as if attempting to touch his/her toes, examiner re-measures the distance between the top and bottom line (Tousignant et al., 2005).

### 2.7 Statistical analysis

Descriptive statistics (mean ± standard deviation) were calculated to determine participants' demographic and clinical characteristics. Baseline characteristics were compared between groups using independent *t*-tests for continuous variables and chi-squared tests for categorical variables; all model assumptions were checked before analysis.

A mixed-effect model was used for analysis utilizing within-subject factor “Time” (pre-intervention vs. post-intervention) as a fixed effects and between subjects factor “Group” [active (*n* = 20) vs. control (*n* = 20)] as a fixed effect, as well as, their interaction (Group x Time). This model was applied for both primary and secondary outcomes, fitted with the “lme4” package in R. Degrees of freedom for the fixed effects were estimated using the Satterthwaite approximation, which provides more accurate significance testing by accounting for variability in smaller samples, thus minimizing the risk of Type I errors. The fit of the models was evaluated using marginal *R*^2^ (representing the variance explained by the fixed effects) and conditional *R*^2^ (representing the variance explained by both fixed and random effects).

All statistical analyses were performed in *R* (Rodrigues et al., 2020). The significance level was set at 0.05, and 95% confidence intervals were calculated for each of the fixed effects.

## 3 Results

### 3.1 Participant characteristics

A total of *n* = 40 participants were enrolled in the study, with n = 20 participants randomly assigned to the active ATT arm and *n* = 20 to the control arm. The mean age of the participants was 32.18 years (SD = 14.52), with no significant differences between active vs. control ATT in gender distribution across groups (53% male vs. female 47%, *p* = 0.752), pain (MPQ: 54.45 ± 12.54 vs. 50.00 ± 8.84, *p* = 0.203), general health (GHQ-12: 18.55 ± 2.46 vs. 20.25 ± 3.43, *p* = 0.081) and disability (RMDQ: 7.35 ± 3.83 vs. 6.35 ± 3.88, *p* = 0.417; Table 1).

**Table 1:** Demographics and clinical features.

**Feature**	**Full sample (*N* = 40)**	**Active ATT (*n* = 20)**	**Sham ATT (*n* = 20)**	***P*-value**
Age (years), mean (SD)	32.18 (14.52)	34.80 (16.03)	29.55 (12.69)	0.258
Sex % male (*n*)	53% (21)	55.0% (11)	50.0 (10)	0.752
McGill pain, mean (SD)	52.23 (10.94)	54.45 (12.54)	50.00 (8.84)	0.203
GH12, mean (SD)	19.40 (3.07)	18.55 (2.46)	20.25 (3.43)	0.081
Roland Morris, mean (SD)	6.85 (3.84)	7.35 (3.82)	6.35 (3.88)	0.417

### 3.2 Primary outcome: pain reduction

**Figure 4:**
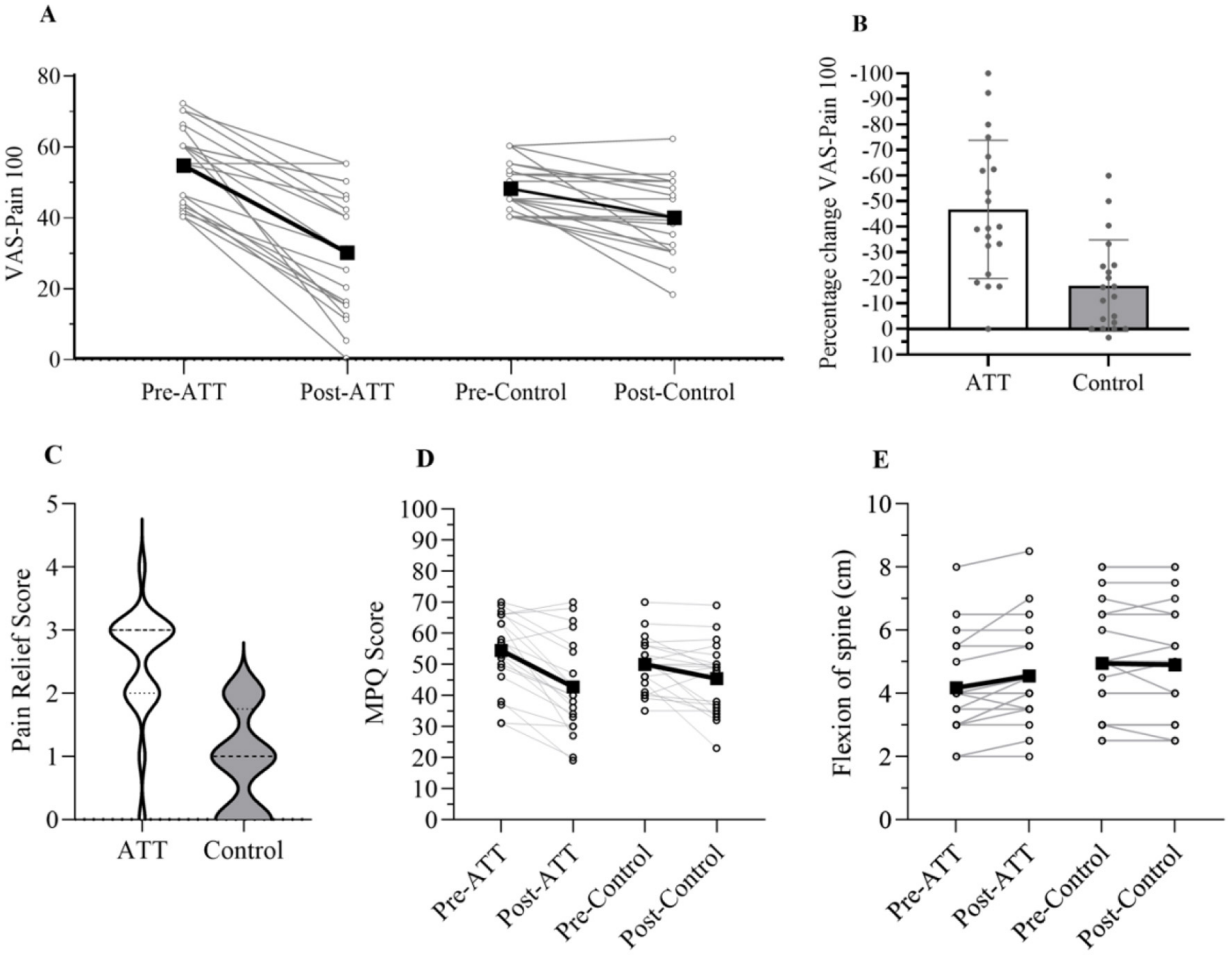
Differences in outcome measures before and after intervention by groups. **(A)** Pairwise comparison of means for the pre-intervention vs. post-intervention VAS-P100 score with individual data points overlaid. **(B)** Mean percentage change in VAS-P100 scores within subjects for ATT and control conditions. **(C)** Comparison of 5-Point Verbal Rating Scale for Pain Relief scores assessed post-intervention between ATT and control groups. **(D)** Pairwise comparison of means for the pre-intervention vs. post-intervention MPQ scores with individual data points overlaid. **(E)** Pairwise comparison of means for the pre-intervention vs. post-intervention MMST scores with individual data points overlaid.

The primary outcome, pain intensity as measured by the 100-mm VAS-P, demonstrated significant reductions in pain levels within both the active and control ATT groups (Figure 4A). The mixed-effects model (Table 2) revealed a significant Group × Time interaction (β = 16.40, *p* = 7.88 × 10^−5^), corresponding to a large effect size (Cohen's *d* = 1.44). This indicates that the active ATT intervention was substantially more effective in reducing pain than the control. On average, VAS-P100 scores decreased by 46.80% in the active condition compared with 17.02% in the control condition (Figure 4B).

**Table 2:** Mixed effects model summary of VAS-P 100.

**Fixed effects**	**Estimate (β)**	**Std. error**	**d*f***	***t*-value**	***p*-value**	**95% CI**
Intercept	54.750	2.730	58.887	20.054	<2.0e−16^***^	(49.452, 60.048)
Group	−6.550	3.861	58.887	−1.696	0.0951	(−14.043, 0.943)
Time	−24.600	2.621	38.000	−9.385	1.93e−11^***^	(−29.730, −19.470)
Group: time	16.400	3.707	38.000	4.424	7.88e−05^***^	(9.145, 23.655)
Variance (intercept): 80.36 (95% CI: [5.935, 11.969])						
Residual variance: 68.71 (95% CI: [6.587, 10.234])						
Model fit: (0.365, 0.707)						

^***^p < 0.001.

### 3.3 Secondary outcomes

Participants in the active ATT on average reported a 22% reduction in pain according to MPQ scores, while the control group reported an average of 9% reduction (Figure 4D). The mixed-effects model revealed a significant effect of Group × Time interaction (β = 7.200, *p* = 0.0164; Table 3). On the 5-point verbal pain relief scale (Figure 4C), the active ATT group reported significantly higher pain-relief scores (mean = 2.50, SD = 0.89) than the control group (mean = 0.90, SD = 0.79; *t*(38) = 6.025, *p* = 5.263 × 10^−7^). Anxiety levels, as measured by the STAI, were analyzed in two parts, the “state” (STAI-S) and “trait” (STAI-T) measures. STAI-S decreased over time in both groups, with a 12.79% reduction compared to an 9.80% reduction in the control group; however, no significant Group × Time interaction was found (*p* = 0.4089; Table 3). The STAI-T scores improved by 7.56% in the active ATT group and 4.54 % in the control group, though no significant Group × Time interaction was observed (*p* = 0.3777). The MMST, assessing lumbar flexibility, showed significant improvement in the active group when compared to the control group. The Group × Time interaction (β = −0.425, *p* = 0.0031, 95 % CI [−0.69, −0.16]) indicated that the active ATT group achieved a significantly greater degree of lumbar flexion than the control group. Mean MMST values increased from 4.18 ± 1.52 cm pre-intervention to 4.55 ± 1.57 cm post-intervention in the active group, compared with 4.95 ± 1.84 cm to 4.90 ± 1.88 cm in the control group (Figure 4E).

**Table 3:** Mixed effects model summary of secondary outcomes.

**Fixed effects**	**Estimate (β)**	**Std. error**	**d*f***	***t*-value**	***p*-value**	**95% CI**
**McGill pain questionnaire**						
Intercept	54.550	2.744	49.702	13.748	<2.0e−16^***^	(49.109, 59.791)
Group	−4.450	3.881	49.702	−1.147	0.2570	(−12.004, 3.104)
Time	−11.750	2.026	38.000	−5.799	1.08e−06^***^	(−15.716, −7.784)
Group: time	7.200	2.866	38.000	−2.512	0.0164^*^	(1.591, 12.809)
Variance = 109.57 (95% CI: [7.829, 13.384])						
Residual variance: 41.06 (95% CI: [5.092, 7.911])						
Model fit = (0.119, 0.760)						
**State-trait anxiety inventory-state**						
Intercept	34.750	1.989	53.158	17.467	<2.0e−16^***^	(30.883, 38.617)
Group	1.000	2.813	53.158	0.355	0.72367	(−4.469, 6.469)
Time	−5.850	1.651	38.000	−3.543	0.00107^**^	(−9.082, −2.618)
Group: time	1.950	2.335	38.000	0.835	0.4089	(−2.620, 6.520)
Variance = 51.89 (95% CI: [5.216, 9.339])						
Residual variance = 27.27 (95% CI: [4.149, 6.447])						
Model fit = (0.084, 0.684)						
**State-trait anxiety inventory-trait**						
Intercept	37.750	2.484	42.923	15.195	<2.0e−16^***^	(32.901, 42.599)
Group	3.950	3.513	42.923	1.124	0.26715	(−2.908, 10.808)
Time	−3.500	1.228	38.000	−2.850	0.00702^**^	(−5.903, −1.097)
Group: time	1.550	1.737	38.000	0.893	0.3777	(−1.849, 4.949)
Variance = 108.36 (95% CI: [8.114, 13.021])						
Residual variance = 15.08 (95% CI: [3.086, 4.794])						
Model fit = (0.059, 0.885)						
**Modified-modified Schöber test**						
Intercept	4.1750	0.3820	39.1981	10.929	1.83e−13^***^	(3.428, 4.922)
Group	0.7750	0.5403	39.1981	1.434	0.159369	(−0.282, 1.832)
Time	0.3750	0.0952	38.000	3.939	0.000338^***^	(0.189, 0.561)
Group: time	−0.425	0.1346	38.000	−3.157	0.003119^**^	(−0.688, −0.162)
Variance = 2.828 (95% CI: [1.331, 2.083])						
Residual variance = 0.091 (95% CI: [0.239, 0.372])						
Model fit = (0.033, 0.970)						

^*^p < 0.05, ^**^p < 0.01, ^***^p < 0.001.

### 3.4 Tolerability and feasibility

All participants completed the 40-min ATT or control session without reporting any adverse events. In accordance with trial protocol, two participants in the ATT group requested a reduction in heat intensity, which was adjusted accordingly, allowing them to complete the intervention as planned.

## 4 Discussion

The results of this randomized, double-blind, controlled trial support the efficacy of a single session of thermo-mechanical therapy in significantly reducing pain intensity in individuals with chronic non-specific lower back pain (Maher et al., 2017; Buchbinder et al., 2018). Our analysis revealed that participants in the active group reported clinically significant analgesic effects (Thompson et al., 2020; Jiang et al., 2022). All primary and secondary outcomes related to pain (VAS-P100, MPQ, and VRS) provided data supporting active ATT's efficacy in reducing pain levels (Buselli et al., 2011; Kim et al., 2020). In addition to improvements in pain, participants who received the active treatment had significant improvements in mobility (MMST) compared to the control group (Chen et al., 2015; Nadler, 2015). These findings are consistent with prior literature on the effects of thermal and mechanical stimulation on analgesic cellular mechanisms, musculoskeletal pain, and quality of life (Hardy and Woodall, 1998; Freiwald et al., 2021; Green, 2004). Trend in STAI scores may be accounted for in part by the relaxation that occurs from laying down for 36 min.

Due to the synergistic effects of heat and mechanical stimulation, combined thermo-mechanical therapy by ATT may offer superior pain relief compared to mechanical or thermal therapies in isolation (Kim et al., 2022; Choi et al., 2020a). Although physiological mechanisms of analgesia and therapeutic benefit for both mechanical and thermal therapies are well validated, physiological mechanisms for synergy of pain relief in combined thermal and mechanical therapy remain elusive. However, deforming tissue to decrease the distance between thermal actuators and target tissue significantly increases the dose of thermal energy that reaches the target tissue. In addition, it is well documented that heated tissues are more malleable and, as such, allows mechanical stimulation to have greater deformation of the tissue which could lead to greater analgesic and vasodilatory effects. Further advantages of the ATT system include: (1) a non-drug, minimal-risk intervention (Lee et al., 2011); (2) providing consistent, standardized treatment doses across participants (practitioner-based variability in manual therapies), which in turn (3) supports RCT rigor (Tiidus and Shoemaker, 1995), (4) device deployability, including to professional (e.g., rehabilitation) or home settings; and (5) supporting both responsive and prophylactic uses (Sefton et al., 2010; Rodrigues et al., 2020). Our RCT provides proof-of-concept evidence supporting the efficacy of ATT-delivered thermo-mechanical therapy for pain management, rehabilitation, and regulatory dysfunctions and warrants further investigation before clinical implementation (Crane et al., 2012; Choi et al., 2020b; Krause et al., 2000).

## Data Availability

The raw data supporting the conclusions of this article will be made available by the authors, without undue reservation.
